# A Standardized Patient Experience: Elevating Interns to Expected Level of Clinical Competency

**DOI:** 10.5811/westjem.2020.10.49092

**Published:** 2020-11-20

**Authors:** Megan Cifuni, Caroline Stoddard, Scott Witt, Camiron Pfennig-Bass, Mark Pittman

**Affiliations:** Prisma Health Upstate, Department of Emergency Medicine, Greenville, South Carolina

## Abstract

**Introduction:**

Medical students transition to intern year with significant variability in prior clinical experience depending on their medical school education. This leads to notable differences in the interns’ ability to perform focused histories and physical exams, develop reasoned differentials, and maximize care plans. Providing a foundational experience for these essential skills will help to establish standardized expectations despite variable medical school experiences.

**Methods:**

During an orientation block, interns participated in a standardized patient experience. Interns were presented with three common chief complaints: abdominal pain; chest pain; and headache. Faculty observed the three patient encounters and provided immediate verbal and written feedback to the interns based on a standardized grading rubric.

**Results:**

All residents that participated “agreed” or “strongly agreed” that the experience was a meaningful educational experience. 90% of the interns reported the experience would change their clinical practice. Additionally, 75% of residents survyed one year after the experience felt the experience changed their clinical practice. Faculty felt the learning experience allowed them to address knowledge gaps early and provide early guidance where needed.

**Conclusion:**

This article describes an emergency medicine residency program’s effort to provide a foundational experience for interns in evaluating emergency department patients. The intent was to “level the playing field” and establish “good habits” early in intern year with the realization that prior experiences vary significantly in July of intern year.

## BACKGROUND

Since the 2008 development of the Emergency Medicine Milestones Project, residency programs have focused on using these competency-based benchmarks to assess resident progression through training. The stepwise progression of the Milestones acknowledges that medical training is certainly a continuum of learning.^1^,^2^ We have found that new emergency medicine (EM) interns fall at variable points on this spectrum of medical knowledge and skill. However, it is the assumption of the Accreditation Council for Graduate Medical Education that graduating medical students, and thus new interns, function at a Milestone Level 1.^3^,^4^ It is our experience that diverse medical school experiences lead to some interns beginning EM residency not yet able to achieve this Level 1 competency.

Determining which interns require more directed guidance and focused education can be difficult in a busy clinical environment. Direct observation is often cited as a tool to assess clinical competency.^5^ However, we find that direct observation can be difficult and is often underused in hectic emergency department (ED) environments. We propose a uniform assessment of incoming EM interns in a mock standardized patient scenario with the purpose of gauging gaps in clinical competencies. This allows for more directed education and focused correction of potential knowledge gaps.

## OBJECTIVES

The objective of creating a standardized patient experience for incoming EM interns is multifold. Our first goal was to simulate realistic EM patient encounters focusing on common ED chief complaints. Our second goal was to observe the EM interns interacting with a standardized patient and determine potential correctable behaviors during history taking and physical examination. The final goal was to evaluate the interns’ ability to synthesize the patient presentation, provide a reasoned differential and plan, and communicate effectively with the patient.

## CURRICULAR DESIGN

We created a simulated patient encounter for three common ED chief complaints: chest pain; abdominal pain; and headache. Standardized patients were briefed and prepared on their cases prior to the encounters. To begin the encounters, interns were informed of the patient’s age and gender, chief complaint, and vital signs. Interns entered an exam room and were given 10 minutes to perform a focused history and physical exam. During this time, the interns were observed by a faculty member (assistant program director or medical education fellow). The interns were then given 15 minutes to collect their thoughts and present the patient to the faculty member. Through this presentation, they were expected to develop and communicate a reasoned differential and plan of care for the patient. The interns were then assessed using a rubric created to identify potential gaps in their knowledge base and skill set.

We were able to assess several of the Level 1 Milestones for patient care (PC) during this activity:

- Performs and communicates a reliable, comprehensive history and physical exam (PC2)
- Determines the necessity of diagnostic studies (PC3)
- Constructs a list of potential diagnoses based on chief complaint and assessment (PC4)
- Describes basic resources available for care of the emergency department patient (PC7).

**Figure 1:**
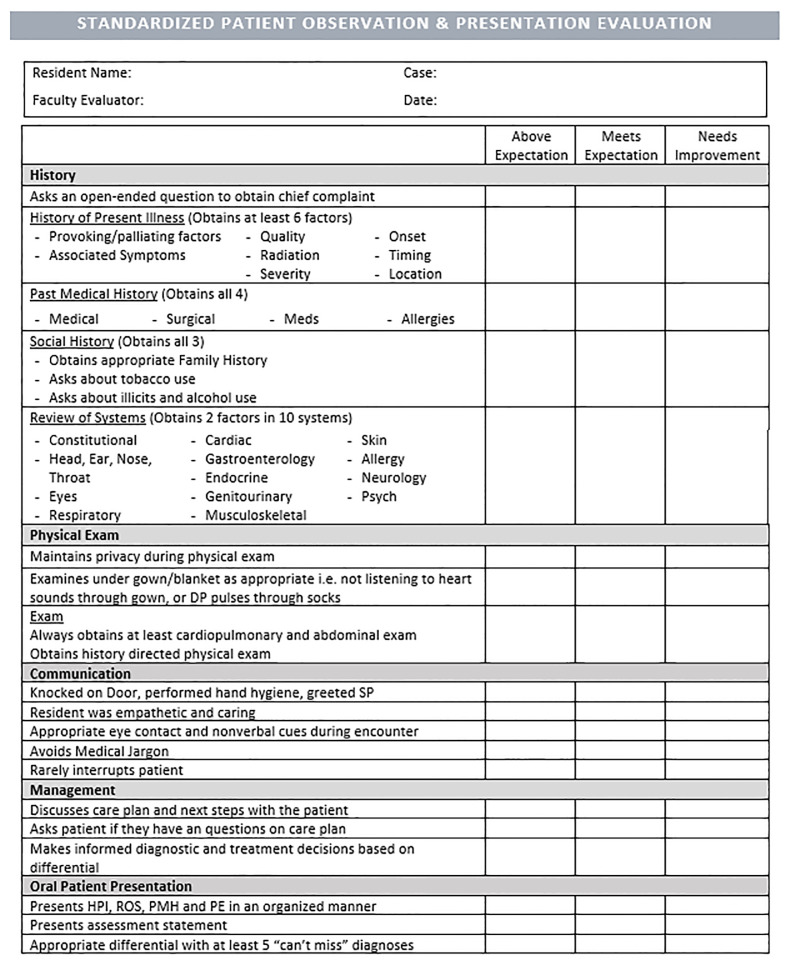
Page one of the standardized patient rubric to assess interns’ skills in patient assessment.

**Figure 2:**
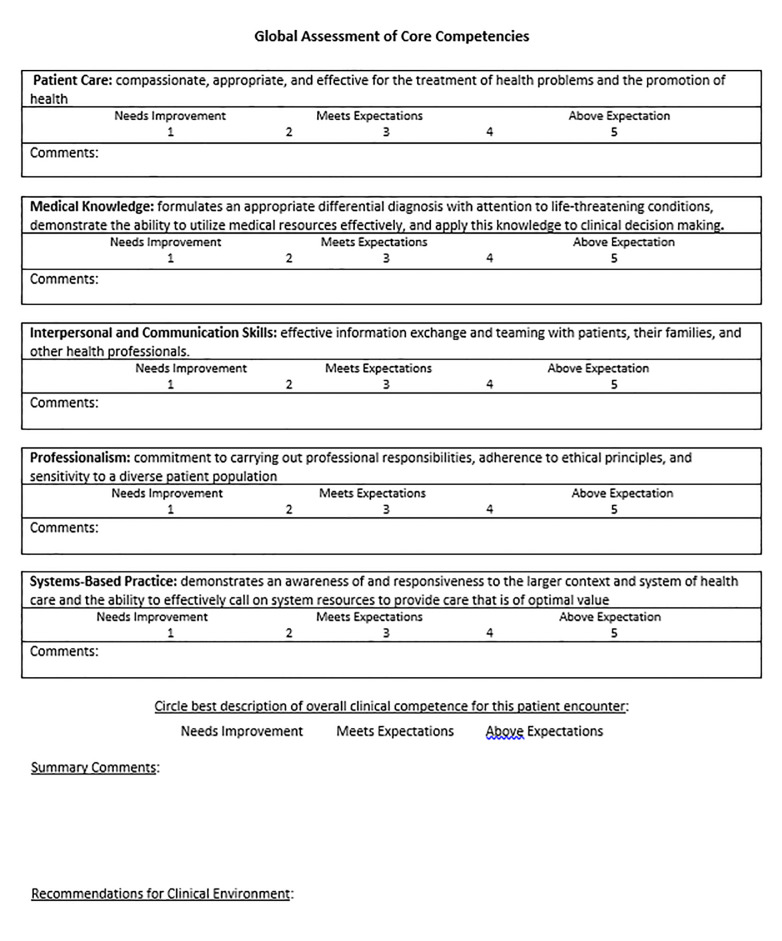
Page two of the standardized patient rubric.

Based on the above milestones and our judgment of essential EM intern skills in patient assessment, we created an assessment rubric (Figures 1 and 2).

The first year we implemented this activity, interns completed a documentation chart after their interaction with the patient as opposed to presenting the patient orally to the observing faculty member. However, we found that review of the written documentation was very labor intensive and time-consuming and therefore delayed assessment by the faculty member, prohibiting immediate, timely feedback. The oral presentations allow for the faculty member to directly provide verbal and then written feedback to the interns without delay. For other programs considering implementation but worried about cost, we used a total of six standardized patients at $20 per hour for a total of five hours. The observation certainly could use additional faculty or residents to play the part of standardized patients for a similar effect.

## IMPACT/EFFECTIVENESS

The implementation of this standardized patient experience during orientation month has been well received by our residents. Immediately following the activity, 100% of interns surveyed “agreed” or “strongly agreed” that it was a meaningful and educational experience; 90% of those interns also reported that the encounter would change their clinical practice starting intern year. These findings have persisted over time as 90% of our second-year residents, who were surveyed one year after the experience, also agreed that the activity was meaningful and educational, and 75% felt that the experience had changed their clinical practice.

This standardized patient encounter allows educational faculty to address any knowledge gaps or re-direct any poor habits early in intern year. We have found that although direct clinical observation is commonly cited as a tool for assessing residents, it is often cumbersome on shift and therefore done hastily or infrequently. Creating a standardized patient experience outside of the clinical arena allows for more careful observation and meaningful feedback. These initial assessments can then be compared to on-shift direct observations completed by faculty to determine intern progression. This innovation could certainly be adopted by other EM programs or even by other specialties as a benchmark assessment and intervention to help “level the playing field” at the start of intern year.
